# Thigh Circumference and Diabetes: Obesity as a Potential Effect Modifier

**DOI:** 10.2188/jea.JE20120174

**Published:** 2013-09-05

**Authors:** Keum Ji Jung, Heejin Kimm, Ji Eun Yun, Sun Ha Jee

**Affiliations:** 1 Department of Epidemiology and Health Promotion, Graduate School of Public Health, Yonsei University, Seoul, Republic of Korea; 2 Department of Rehabilitation Standard & Policy, Korea National Rehabilitation Research Institute, Seoul, Republic of Korea; 3 Institute for Health Promotion, Graduate School of Public Health, Yonsei University, Seoul, Republic of Korea

**Keywords:** thigh circumference, diabetes, effect modifier, obesity

## Abstract

**Background:**

Thigh circumference is associated with diabetes risk; however, the role of obesity as a potential effect modifier has not been well studied.

**Methods:**

We examined the association between thigh circumference and diabetes in a cross-sectional study of 384 612 Koreans aged 30 to 79 years. The association between diabetes and thigh circumference in relation to body mass index (BMI) was analyzed among 315 628 participants, using multivariate logistic regression. Thigh circumference was categorized into 9 percentile categories—namely, the 2.5th, 5th, 10th, 25th, 50th, 75th, 90th, 95th, and 97.5th percentiles—and the 50th percentile was used as the reference value for thigh circumference. Separate analyses were performed for men and women.

**Results:**

The association of thigh circumference with diabetes showed contradictory patterns before and after adjustment for BMI and waist circumference. Small thigh circumference was associated with greater risk of diabetes among men and women. This relationship was stronger among participants younger than 50 years, although age was not a significant effect modifier. BMI was a significant effect modifier among men with a BMI of less than 25 kg/m^2^. Among women, diabetes risk increased with smaller thigh circumference.

**Conclusions:**

Small thigh circumference was associated with diabetes, and this association was stronger among participants with a BMI of less than 25 kg/m^2^. Thigh circumference might be a useful diabetes marker in lean populations.

## INTRODUCTION

Type 2 diabetes is the leading preventable cause of cardiovascular disease and premature death worldwide.^1^^,^^2^ The prevalence and incidence of diabetes are increasing rapidly in both developed and developing countries.^3^^,^^4^ Moreover, the prevalence of type 2 diabetes is rapidly increasing in Asia, due in part to increasing obesity.^5^

Imaging studies suggest that Asians have greater visceral adiposity than whites at all body mass index (BMI) values and hence a higher risk for type 2 diabetes.^5^ Waist and hip circumference were found to have independent and opposite associations with potential cardiovascular hazard factors among white men and women.^6^ Stratification by BMI tertiles revealed that the association of waist circumference with abdominal subcutaneous fat was stronger for a group with higher BMI values.^6^

A small thigh circumference has also been implicated as a causal risk factor for multiple diseases,^6^^–^^9^ and the number of studies investigating thigh circumference as a useful indicator of body fat has increased substantially.^10^^,^^11^ Recent studies suggest that smaller thighs are disadvantageous to health and survival^7^ and increase diabetes risk^6^^,^^12^ among both sexes. However, in some studies, smaller thighs were related to low muscle mass.^13^^,^^14^ Potential differences with respect to age and BMI in the relationship of thigh circumference to glucose metabolism have not been reported.

We hypothesized that age and obesity modify the association between thigh circumference and diabetes and thus evaluated this association, in relation to age and BMI, among healthy Korean men and women.

## METHODS

### Study population

The study population consisted of 384 612 individuals who participated in the Korea Medical Institute (KMI) Study and had routine health examinations at the KMI between January 2009 and December 2011. The KMI is a health examination service provider. Because all employed people are legally required to undergo a biannual medical checkup in Korea, and companies must provide this service to their workers, most examinees at the KMI were workers. They were informed of the purpose and content of the present research. The Yonsei University Institutional Review Board on Human Research approved this study.

To avoid confounding of the association between thigh circumference and diabetes by pre-existing disease, 6738 subjects who reported having cardiovascular diseases (*n* = 2491), stroke (*n* = 810), or any cancer (*n* = 3575) were excluded. In addition, 888 subjects with missing information on thigh circumference, BMI, waist circumference, fasting serum glucose, serum lipids, smoking status, or exercise, and those with an extremely low BMI (<14.0) or short stature (≤1.3 m) were excluded. The final sample included 315 628 subjects aged 30 to 79 years. Among them, 47 137 participants who consented to collection of blood samples and completed the informed consent forms were included in the analysis.

### Data collection and assays

Self-reported alcohol consumption, smoking status, and physical activity level were estimated from the questionnaire. During a standardized examination at KMI, participants were asked if they had ever smoked or if they exercised regularly, using a standardized health questionnaire. Information was also collected on demographic characteristics such as age, sex, family history of diabetes, cigarette smoking status (never-smoker, ex-smoker, or current-smoker), and alcohol consumption status (nondrinker and ever-drinker). Current smokers and ex-smokers were asked to report the average number of cigarettes they smoke or had smoked per day. Waist circumference was measured midway between the lower rib and iliac crest. Thigh circumference was measured on the left leg directly below the gluteal fold, while participants wore the same type of hospital gowns used during the health check-up. Thigh circumference was measured once. The correlation coefficients for intra- and inter-technician reliability were 0.971 and 0.957, respectively.

A registered nurse or blood pressure technician used a standard mercury sphygmomanometer to measure blood pressure while the participants were seated. Systolic and diastolic blood pressure were measured after a minimum rest period of 5 minutes. Blood pressure was measured twice if it was higher than 120/80 mm Hg.

For clinical chemistry assays, serum was separated from peripheral venous blood samples obtained from each participant after 12 hours of fasting. Fasting blood glucose, total cholesterol, triglycerides, and high-density lipoprotein cholesterol (HDL-C) were measured using a Hitachi-7600 analyzer (Hitachi Ltd., Tokyo, Japan). BMI was calculated as weight (kg) divided by the square of the height (m^2^). All measurements were performed by the central laboratory, located at the KMI Seoul North site. Data quality control was maintained in accordance with the procedures of the Korean Association of Laboratory Quality Control.

Diabetes mellitus was defined as a fasting blood glucose of at least 126 mg/dL (7.0 mmol/L) or self-reported treatment for diabetes.^15^ In the questionnaire used for this present study, participants were asked if they were taking any medication for treatment of diabetes. If so, they were asked to write down the name of the medication.

### Statistical analysis

Data were expressed as mean (SD). Multiple logistic regression models were used to assess the independent association of thigh circumference with type 2 diabetes. Separate analyses were performed for men and women. We fitted 2 models examining the association between thigh circumference and diabetes, using different adjustment schemes. The first model (basic model) included age, smoking status, physical activity, and family history of diabetes. Models 2, 3, and 4 were additionally adjusted for waist circumference and/or BMI, to evaluate the effects of those variables on the association. In all analyses, the thigh circumference was categorized into 9 percentile categories (2.5th, 5th, 10th, 25th, 50th, 75th, 90th, 95th, and 97.5th percentiles), to allow for the possibility of nonlinear associations. The 50th percentile was used as the reference value for thigh circumference.

Odds ratios (ORs) and 95% CIs were calculated for a 1-SD increase in thigh circumference (on a continuous scale), with SD defined as the square root of the variance. The interactions between age and thigh circumference, BMI and thigh circumference, and waist circumference and thigh circumference were tested by inserting first-order interaction terms into regression models using the likelihood ratio χ^2^. In logistic regression analysis consisting of age, BMI, waist circumference, and thigh circumference, the area under the receiver operating curve (AUC) plus 95% CI was used to evaluate the overall ability of thigh circumference to discriminate diabetes status. All analyses were conducted using SAS statistical software version 9.2 (SAS Institute Inc., Cary, NC, USA). All statistical tests were 2-sided, and the null hypothesis of no difference was rejected if *P*-values were less than 0.05 or if 95% CIs for the ORs did not include 1.

## RESULTS

Overall mean age was 42.3 years (42.6 years for men and 41.9 years for women), and mean BMI was 23.6 kg/m^2^ (24.5 kg/m^2^ for men and 22.2 kg/m^2^ for women). Overall prevalence of type 2 diabetes was 4.6% (5.7% for men and 2.7% for women). Overall mean thigh circumference was 53.2 cm (54.3 cm for men and 51.5 cm for women). The correlation between hip circumference and thigh circumference among 141 participants was 0.71 (0.82 for men and 0.49 for women).

As shown in Table 1, mean age, BMI, waist circumference, fasting serum glucose, systolic blood pressure, and triglyceride values were higher among patients with diabetes than among nondiabetic patients. In addition, participants with diabetes exercised more.

**Table 1.** General characteristics of study participants

	Men		Women	
		
	Diabetes^a^ *n* = 11 386	No diabetes *n* = 188 037	Diabetes^a^ *n* = 3129	No diabetes *n* = 113 076
Age, years	49.9 (9.8)	42.2 (8.7)	54.4 (11.5)	41.6 (9.5)
Body mass index	26.0 (3.2)	24.4 (2.9)	25.3 (3.8)	22.1 (3.1)
Thigh circumference, cm	53.9 (5.3)	54.3 (4.8)	51.8 (5.7)	51.4 (4.8)
Waist circumference, cm	88.4 (8.1)	84.3 (7.6)	83.6 (9.4)	73.7 (8.1)
Fasting serum glucose, mg/dL	153.3 (46.2)	93.0 (10.0)	145.8 (45.8)	89.6 (9.3)
Systolic blood pressure, mm Hg	126.1 (13.5)	121.8 (12.2)	123.7 (14.8)	112.1 (13.0)
Total cholesterol, mg/dL	197.4 (41.1)	197.9 (33.5)	199.6 (41.3)	188.2 (32.9)
Triglyceride, mg/dL	216.2 (167.5)	155.7 (105.6)	158.0 (101.0)	95.4 (58.8)
Family history of diabetes (yes)	24.6	9.6	27.2	11.6
Cigarette smoking (current)	34.0	28.8	1.8	3.2
(ex)	43.4	43.1	3.0	3.5
Exercise (none)	22.8	25.5	37.2	44.7

^a^Diabetes was defined as a fasting serum glucose of ≥126 mg/dL or history of diabetes treatment.

Data are means (SD) or *n* (%).

The association of thigh circumference with diabetes substantially changed after adjustment for BMI and waist circumference. In model 4, smaller thigh circumference was associated with diabetes among men and women. In model 4, all CIs for each percentile category were statistically significant among men (Table 2) and women (Table 3).

**Table 2.** Odds ratio (95% CIs) for the association between thigh circumference and diabetes among 199 423 men aged 30–79 years

Percentile of thigh circumference (cm)	Model 1	Model 2	Model 3	Model 4
2.5 (<45)	0.81 (0.72–0.91)	0.98 (0.87–1.11)	2.11 (1.85–2.39)	2.07 (1.82–2.35)
5 (45–<47)	0.93 (0.83–1.03)	1.07 (0.96–1.19)	1.83 (1.64–2.04)	1.81 (1.62–2.02)
10 (47–<48)	1.04 (0.93–1.17)	1.17 (1.05–1.31)	1.82 (1.62–2.05)	1.80 (1.60–2.03)
25 (48–<51)	0.94 (0.89–1.00)	1.02 (0.96–1.08)	1.38 (1.30–1.47)	1.37 (1.29–1.46)
50 (51–<57)	1.00	1.00	1.00	1.00
75 (57–<60)	1.15 (1.08–1.21)	1.04 (0.98–1.10)	0.78 (0.74–0.83)	0.78 (0.74–0.83)
90 (60–<62)	1.22 (1.12–1.33)	0.97 (0.89–1.06)	0.65 (0.59–0.71)	0.64 (0.59–0.70)
95 (62–<64)	1.54 (1.38–1.71)	1.01 (0.90–1.13)	0.66 (0.59–0.74)	0.63 (0.57–0.71)
97.5 (≥65)	1.98 (1.79–2.20)	0.81 (0.72–0.93)	0.58 (0.52–0.65)	0.52 (0.46–0.59)

Per 1-SD increase	1.16 (1.13–1.18)	0.98 (0.95–1.00)	0.72 (0.71–0.74)	0.72 (0.70–0.74)

AUC	0.763 (0.758–0.767)	0.789 (0.785–0.793)	0.793 (0.789–0.797)	0.795 (0.791–0.798)

Abbreviation: AUC, area under the curve.

Model 1: adjusted for age, smoking, exercise, and family history of diabetes.

Model 2: model 1 + additional adjustment for body mass index.

Model 3: model 1 + additional adjustment for waist circumference.

Model 4: model 1 + additional adjustment for body mass index and waist circumference.

**Table 3.** Odds ratio (95% CIs) for the association between thigh circumference and diabetes among 116 205 women aged 30–79 years

Percentile of thigh circumference (cm)	Model 1	Model 2	Model 3	Model 4
2.5 (<43)	1.12 (0.90–1.39)	1.39 (1.12–1.73)	2.61 (2.09–3.26)	2.59 (2.07–3.24)
5 (43–<44)	1.24 (0.95–1.62)	1.47 (1.12–1.91)	2.28 (1.72–3.00)	2.27 (1.72–2.99)
10 (44–<46)	1.18 (1.01–1.38)	1.34 (1.15–1.57)	1.84 (1.65–2.29)	1.94 (1.65–2.28)
25 (46–<48)	1.05 (0.92–1.20)	1.15 (1.01–1.31)	1.50 (1.31–1.71)	1.49 (1.30–1.71)
50 (48–<54)	1.00	1.00	1.00	1.00
75 (54–<57)	1.24 (1.12–1.38)	1.08 (0.97–1.20)	0.83 (0.74–0.93)	0.83 (0.74–0.92)
90 (57–<59)	1.43 (1.23–1.66)	1.09 (0.94–1.28)	0.72 (0.62–0.85)	0.72 (0.61–0.84)
95 (59–<61)	1.77 (1.48–2.11)	1.15 (0.95–1.39)	0.71 (0.59–0.86)	0.69 (0.57–0.83)
97.5 (≥61)	2.55 (2.17–3.00)	0.82 (0.66–1.02)	0.55 (0.45–0.66)	0.48 (0.40–0.59)

Per 1-SD increase	1.19 (1.15–1.24)	0.93 (0.89–0.97)	0.72 (0.69–0.76)	0.70 (0.67–0.74)

AUC	0.832 (0.825–0.839)	0.868 (0.862–0.874)	0.871 (0.866–0.778)	0.874 (0.868–0.880)

Abbreviation: AUC, area under the curve.

Model 1: adjusted for age, smoking, exercise, and family history of diabetes.

Model 2: model 1 + additional adjustment for body mass index.

Model 3: model 1 + additional adjustment for waist circumference.

Model 4: model 1 + additional adjustment for body mass index and waist circumference.

Tables 4 and 5 show the ORs for thigh circumference, in our analysis of age and BMI as effect modifiers. The association between thigh circumference and diabetes was weaker among older age groups. The AUC was higher among participants with a low BMI. Thus, the association between thigh circumference and diabetes was significantly stronger among thin participants than among obese participants (Tables 4 and 5). The association between thigh circumference and diabetes was further evaluated by stratifying BMI according to World Health Organization (WHO) definitions (eTables 1 and 2).

**Table 4.** Odds ratio (95% CIs) for the association between thigh circumference and diabetes among 199 423 men aged 30–79 years

Percentile of thigh circumference (cm)	Age, years			Body mass index, kg/m^2^		
	
	<50 (5,96/158 149)	50–64 (4453/36 168)	65+ (1037/5106)	<23 (2272/62 291)	23–24.9 (2923/56 399)	25+ (6191/80 733)
2.5 (<43)	3.43 (2.70–4.34)	2.55 (2.13–3.07)	1.62 (1.21–2.17)	2.79 (2.33–3.33)	1.94 (1.42–2.65)	1.08 (0.61–1.92)
5 (43–<44)	1.98 (1.61–2.43)	2.19 (1.87–2.56)	1.58 (1.21–2.08)	2.20 (1.87–2.58)	1.87 (1.50–2.34)	1.67 (1.19–2.33)
10 (44–<46)	1.99 (1.62–2.44)	2.12 (1.80–2.50)	1.37 (1.01–1.86)	2.18 (1.84–2.58)	2.06 (1.67–2.56)	1.20 (0.85–1.71)
25 (46–<48)	1.49 (1.36–1.64)	1.47 (1.34–1.61)	1.18 (0.97–1.43)	1.56 (1.39–1.74)	1.53 (1.38–1.70)	1.18 (1.04–1.34)
50 (48–<54)	1.00	1.00	1.00	1.00	1.00	1.00
75 (54–<57)	0.74 (0.69–0.80)	0.76 (0.68–0.85)	0.74 (0.54–1.01)	0.66 (0.48–0.89)	0.66 (0.58–0.76)	0.76 (0.70–0.81)
90 (57–<59)	0.60 (0.54–0.67)	0.56 (0.47–0.68)	0.58 (0.30–1.13)	0.66 (0.31–1.42)	0.40 (0.28–0.57)	0.60 (0.54–0.66)
95 (59–<61)	0.59 (0.51–0.67)	0.63 (0.48–0.82)	0.43 (0.16–1.15)	0.68 (0.16–2.82)	0.57 (0.33–0.98)	0.58 (0.51–0.65)
97.5 (≥61)	0.44 (0.39–0.51)	0.67 (0.51–0.89)	0.77 (0.35–1.69)	0.43 (0.06–3.12)	0.75 (0.41–1.37)	0.47 (0.41–0.53)
AUC	0.786 (0.781–0.792)	0.683 (0.674–0.691)	0.658 (0.639–0.676)	0.824 (0.816–0.832)	0.799 (0.791–0.807)	0.755 (0.749–0.761)

Abbreviation: AUC, area under the curve.

Model adjusted for age, smoking, exercise, family history of diabetes, body mass index, and waist circumference.

**Table 5.** Odds ratio (95% CIs) for the association between thigh circumference and diabetes among 116 205 women aged 30–79 years

Percentile of thigh circumference (cm)	Age, years			Body mass index, kg/m^2^		
	
	<50 (1044/92 224)	50–64 (1454/20 073)	65+ (631/3908)	<23 (875/76 989)	23–24.9 (726/19 843)	25+ (1528/19 373)
2.5 (<45)	4.29 (2.59–7.08)	2.55 (1.80–3.64)	2.31 (1.59–3.37)	2.86 (2.15–3.81)	2.26 (1.25–4.11)	0.90 (0.30–2.68)
5 (45–<47)	2.18 (1.07–4.44)	2.66 (1.77–3.99)	2.20 (1.39–3.49)	2.35 (1.64–3.36)	2.55 (1.47–4.44)	1.47 (0.51–4.22)
10 (47–<48)	2.20 (1.59–3.06)	2.30 (1.83–2.89)	1.54 (1.11–2.15)	2.30 (1.85–2.86)	1.80 (1.28–2.52)	1.38 (0.87–2.17)
25 (48–<51)	1.56 (1.21–2.02)	1.61 (1.32–1.95)	1.56 (1.19–2.04)	1.66 (1.37–2.03)	1.37 (1068–1.77)	1.64 (1.23–2.20)
50 (51–<57)	1.00	1.00	1.00	1.00	1.00	1.00
75 (57–<60)	0.62 (0.52–0.75)	0.83 (0.71–0.97)	0.96 (0.72–1.29)	0.64 (0.48–0.86)	0.52 (0.41–0.66)	0.88 (0.76–1.02)
90 (60–<62)	0.48 (0.37–0.61)	0.68 (0.54–0.86)	0.90 (0.57–1.44)	0.49 (0.26–0.92)	0.35 (0.23–0.54)	0.68 (0.56–0.82)
95 (62–<64)	0.42 (0.32–0.55)	0.59 (0.44–0.79)	1.01 (0.55–1.86)	0.10 (0.01–0.75)	0.36 (0.20–0.66)	0.60 (0.49–0.74)
97.5 (≥65)	0.21 (0.15–0.28)	0.45 (0.32–0.62)	0.60 (0.30–1.20)	0.22 (0.03–1.78)	0.51 (0.25–1.04)	0.37 (0.30–0.47)
AUC	0.840 (0.827–0.852)	0.756 (0.743–0.768)	0.690 (0.668–0.713)	0.866 (0.853–0.879)	0.820 (0.804–0.835)	0.772 (0.760–0.783)

Abbreviation: AUC, area under the curve.

Model adjusted for age, smoking, exercise, family history of diabetes, body mass index, and waist circumference.

**Figure 1.**
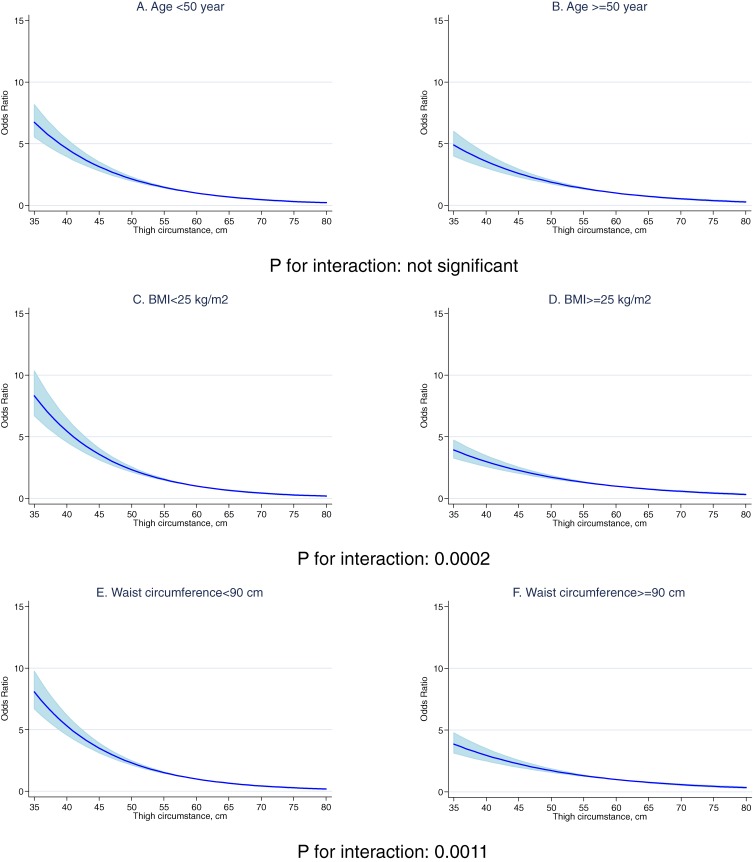
Odds ratio for diabetes associated with thigh circumference in relation to age, body mass index, and waist circumference in men

**Figure 2.**
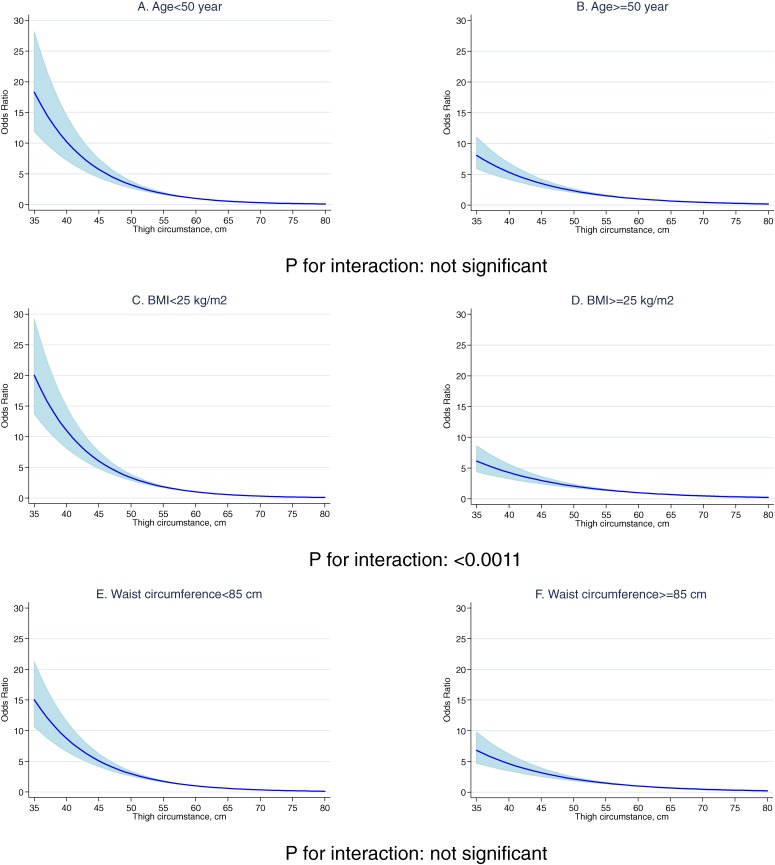
Odds ratio for diabetes associated with thigh circumference in relation to age, body mass index, and waist circumference in women

There was no significant interaction with age (age <50 years vs ≥50 years) among men (Figure 1) or women (Figure 2), which indicates that the association with thigh circumference was similar for younger and older men (A and B in Figure 1) and younger and older women (A and B in Figure 2). However, there was a significant interaction with BMI (BMI <25 kg/m^2^ vs ≥25 kg/m^2^) among men (*P* for interaction: 0.0002) and women (*P* for interaction: <0.0011) (Figures 1 and 2): a BMI of less than 25 was associated with greater diabetes risk.

## DISCUSSION

The results of this large-scale cross-sectional study support the findings of earlier studies,^6^^,^^12^ namely, that smaller thigh circumference was associated with diabetes among men and women. An interaction between thigh circumference and obesity in relation to diabetes was observed: men and women with a BMI of less than 25 kg/m^2^ had a higher risk a diabetes if they had a smaller thigh circumference. In addition, men with a waist circumference of less than 90 cm also had a higher risk of diabetes.

The effect of thigh circumference on diabetes changed after adjustment for BMI and waist circumference. Before adjustment, a smaller thigh circumference was strongly protective for diabetes among men (model 1 in Table 2), and a larger thigh circumference increased the risk of diabetes. However, these associations changed after additional adjustment for BMI (model 2), waist circumference (model 3), and BMI and waist circumference (model 4). The correlation coefficients between thigh circumference and BMI were 0.70 for men and 0.69 for women. The correlation coefficients between thigh circumference and waist circumference were 0.67 for men and 0.60 for women. Therefore, the increased risk of diabetes associated with larger thigh circumference, before adjustment for BMI or waist circumference, may reflect an association between those obesity indicators.

### Comparison with previous findings

Our overall findings are in line with earlier observations. Associations between thigh circumference and diabetes have been reported in several countries. In addition, small thigh circumference has been investigated as a risk factor for diabetes and various health outcomes.^6^^–^^9^ Snijder et al^6^ found that a 1-SD increase in thigh circumference decreased diabetes risk among men (OR = 0.79) and women (OR = 0.64). Our results were similar for both men (OR = 0.68) and women (OR = 0.64). A Danish cohort study, the MONICA project, recently reported that smaller thigh circumference might increase the risk of CVD and early death.^7^ As in other countries, thigh circumference was negatively associated with diabetes risk in Japan.^12^ Other studies have investigated the relations between thigh muscle, metabolic syndrome,^16^^,^^17^ waist-to-thigh ratio,^18^ and type 2 diabetes.^19^

### Obesity as a potential effect modifier

We examined the association between measured thigh circumference and diabetes among more than 300 000 participants; hence, the analysis had sufficient statistical power. To explore interactions between variables, extremely large sample sizes are required. However, most previous cross-sectional and cohort studies had sample sizes that were inadequate for the analysis of such interactions. Therefore, they were unable to carefully investigate interactions of thigh circumference with other factors such as age and obesity indicators. One previous study reported that age did not modify the effect of thigh circumference on total mortality or cardiovascular diseases.^7^ Our results were similar: we found no significant interaction between age groups.

There was a strong interaction between obesity and thigh circumference in our study. BMI was evaluated as a significant effect modifier in men and women, and the association between thigh circumference and diabetes disappeared in overweight participants (BMI ≥25 kg/m^2^) and a thigh circumference less than the 50th percentile. However, a strong positive association remained for lean participants (BMI <25 kg/m^2^) with a thigh circumference less than the 50th percentile. Therefore, participants with a BMI of 25 kg/m^2^ or higher and a thigh circumference less than the 50th percentile might not have a higher risk of diabetes. Those with a BMI greater than 25 kg/m^2^ and a thigh circumference in the 50th percentile or higher may have less diabetes risk.

We reviewed the literature and found no previous stratified analyses of the association of thigh circumference and diabetes risk in relation to age and BMI. Although cross-sectional studies have limitations that warrant consideration, our findings strongly suggest that age and BMI are important modifiers of the association between thigh circumference and diabetes risk.

### Possible mechanism

The distribution of adipose tissue within the thigh is an important body-composition determinant of insulin resistance. Weight loss decreases the amount of adipose tissue in thigh muscle and improves insulin sensitivity in people with obesity or type 2 diabetes mellitus. Our findings suggest that the distribution of adipose tissue in the thigh differs according to BMI. Due to the increase in chronic diseases and loss of body weight among older adults, thigh circumference might not be representative of muscle mass when BMI decreases.

### Study strengths and limitations

The main strength of this study of the association between thigh circumference and diabetes in Korean men and women is its large sample size, which is necessary in examining the possibility of effect modification on some variables. Also, all study variables, including questionnaire and clinical items, were measured at a single laboratory at the KMI.

This study has several limitations. The available data do not allow us to classify participants by diabetes type. However, the proportion of type I diabetes in Korea is low, at 1% of diabetes cases.^20^^,^^21^ Also, the prevalence of type I diabetes was reported to be much lower in Asian countries than in Western countries.^21^ Therefore, most diabetes cases in Korea—about 99%—are likely to be type II diabetes. In addition, the representativeness of the background population was limited because the study participants were young, healthy workers. Despite these limitations, a significant association between thigh circumference and diabetes was found in this study.

Because of the cross-sectional design of the study, we cannot rule out the possibility that smaller thigh circumference may be the result of having diabetes. Also, participants with diabetes exercised more frequently, which might represent a change in lifestyle habits after receiving a diagnosis of diabetes. To avoid the effect of reverse causation and reduce bias, prospective cohort studies are necessary to confirm the association between thigh circumference and diabetes, after excluding participants with diabetes at baseline.

In conclusion, we found that larger thigh circumference was associated with decreased diabetes risk among Korean men and women. A cohort study should examine whether such associations are present among apparently healthy Korean women and other ethnic populations.

## ONLINE ONLY MATERIALS

eTable 1. Odds ratios (95% CIs) for the association between thigh circumference and diabetes among 199,423 men aged 30–79 years.

eTable 2. Odds ratios (95% CIs) for the association between thigh circumference and diabetes among 116,205 women aged 30–79 years.
