# Supplementary material for: Thigh Circumference and Diabetes: Obesity as a Potential Effect Modifier
Source: J Epidemiol. 2013 Sep 5;23(5):329–36. doi: 10.2188/jea.JE20120174 (PMC3775526; doi:10.2188/jea.JE20120174)
Supplement: eTable 1. — Odds ratios (95% CIs) for the association between thigh circumference and diabetes among 199,423 men aged 30–79 years. [file je-23-329-s001.pdf]

eTable 1. Odds ratios (95% CIs) for the association between thigh circumference and diabetes among 199,423 men aged 30-79 years.

	Body mass index, kg/m^2				
	**<21.0**	**21.0-22.9**	**22.9-24.9**	**25.0-27.4**	**27.5+**
2.5 (<43)	3.59 (2.65-4.85)	1.96 (1.51-2.55)	1.93 (1.41-2.63)	1.00 (0.52-1.94)	1.44 (0.45-4.60)
5 (43- <44)	2.34 (1.73-3.16)	2.14 (1.75-2.61)	1.85 (1.48-2.31)	1.64 (1.13-2.37)	1.78 (0.79-4.00)
10 (44- <46)	1.97 (1.40-2.76)	2.27 (1.86-2.78)	2.04 (1.65-2.52)	1.43 (1.00-2.06)	0.22 (0.05-0.96)
25 (46- <48)	1.58 (1.20-2.07)	1.53 (1.35-1.73)	1.52 (1.37-1.68)	1.21 (1.06-1.38)	0.89 (0.63-1.26)
50 (48- <54)	1.00	1.00	1.00	1.00	1.00
75 (54- <57)	0.96 (0.30-3.10)	0.65 (0.47-0.90)	0.67 (0.58-0.77)	0.70 (0.64-0.77)	0.87 (0.78-0.97)
90 (57- <59)	NE	0.69 (0.32-1.49)	0.40 (0.28-0.58)	0.54 (0.46-0.63)	0.69 (0.60-0.78)
95 (59- <61)	NE	0.76 (0.18-3.20)	0.58 (0.34-0.99)	0.50 (0.40-0.64)	0.66 (0.57-0.77)
97.5 (>=61)	NE	0.48 (0.07-3.57)	0.75 (0.41-1.36)	0.59 (0.44-0.79)	0.54 (0.46-0.63)
AUC	0.829 (0.814-0.845)	0.821(0.812-0.831)	0.799(0.791-0.807)	0.757(0.749-0.765)	0.741(0.732-0.751)

Abbreviations: AUC, area under the curve; NE: not estimated due to small sample size.
Adjusted for age, smoking status, exercise, family history of diabetes, and waist circumference.
