# Supplementary material for: Thigh Circumference and Diabetes: Obesity as a Potential Effect Modifier
Source: J Epidemiol. 2013 Sep 5;23(5):329–36. doi: 10.2188/jea.JE20120174 (PMC3775526; doi:10.2188/jea.JE20120174)
Supplement: eTable 2. — Odds ratios (95% CIs) for the association between thigh circumference and diabetes among 116,205 women aged 30–79 years. [file je-23-329-s002.pdf]

eTable 2. Odds ratios (95% CIs) for the association between thigh circumference and diabetes among 116,205 women aged 30-79 years.

| | **Body mass index, kg/m$^2$** | | | | |
|---|---|---|---|---|---|
| | **<21.0** | **21.0-22.9** | **22.9-24.9** | **25.0-27.4** | **27.5+** |
| 2.5 (<43) | 3.31 (2.25-4.85) | 2.45 (1.58-3.80) | 2.16 (1.19-3.92) | 1.27 (0.41-3.87) | NE |
| 5 (43- <44) | 3.28 (2.05-5.26) | 1.69 (0.97-2.95) | 2.48 (1.42-4.32) | 1.12 (0.30-4.11) | 2.43 (0.31-18.9) |
| 10 (44- <46) | 2.36 (1.69-3.29) | 2.42 (1.81-3.25) | 1.77 (1.26-2.48) | 1.19 (0.70-2.04) | 1.88 (0.76-4.65) |
| 25 (46- <48) | 1.62 (1.18-2.23) | 1.82 (1.41-2.34) | 1.37 (1.06-1.77) | 1.50 (1.07-2.09) | 1.96 (1.03-3.71) |
| 50 (48- <54) | 1.00 | 1.00 | 1.00 | 1.00 | 1.00 |
| 75 (54- <57) | 1.19 (0.72-1.98) | 0.51 (0.36-0.72) | 0.53 (0.42-0.67) | 0.88 (0.72-1.07) | 0.90 (0.72-1.14) |
| 90 (57- <59) | 0.75 (0.18-3.06) | 0.42 (0.21-0.87) | 0.37 (0.24-0.56) | 0.64 (0.48-0.86) | 0.71 (0.55-0.93) |
| 95 (59- <61) | 1.19 (0.16-8.71) | NE | 0.38 (0.21-0.69) | 0.62 (0.44-0.89) | 0.58 (0.44-0.77) |
| 97.5 (>=61) | NE | 0.37 (0.05-2.69) | 0.54 (0.26-1.10) | 0.42 (0.27-0.65) | 0.39 (0.30-0.50) |
| | | | | | |
| AUC | 0.852(0.829-0.875) | 0.856(0.840-0.873) | 0.820(0.804-0.835) | 0.771(0.755-0.788) | 0.750(0.733-0.767) |

Abbreviations: AUC, area under the curve; NE: not estimated due to small sample size.
Adjusted for age, smoking status, exercise, family history of diabetes, and waist circumference.
